# UP'S: A Cohort Study on Recovery in Psychotic Disorder Patients: Design Protocol

**DOI:** 10.3389/fpsyt.2020.609530

**Published:** 2021-01-12

**Authors:** Bernice C. van Aken, Ayuk Bakia, André I. Wierdsma, Yolande Voskes, Jaap Van Weeghel, Evelyn M. M. van Bussel, Carla Hagestein, Andrea M. Ruissen, Pien Leendertse, Wishal V. Sewbalak, Daphne A. van der Draai, Alice Hammink, M. E. Mandos, Mark van der Gaag, Annette E. Bonebakker, Christina M. Van Der Feltz-Cornelis, Cornelis L. Mulder

**Affiliations:** ^1^Department of Psychiatry, Erasmus MC, Epidemiological and Social Psychiatric Research Institute, Rotterdam, Netherlands; ^2^Department of Ethics, Law and Humanities, Amsterdam UMC, Amsterdam, Netherlands; ^3^GGz Breburg, Tilburg, Netherlands; ^4^Phrenos Centre of Expertise, Utrecht, Netherlands; ^5^Tranzo Department, Tilburg School of Behavioural and Social Sciences, Tilburg University, Tilburg, Netherlands; ^6^Parnassia Psychosis Research, Den Haag, Netherlands; ^7^GGz Oost Brabant, Oss, Netherlands; ^8^Antes, Rotterdam, Netherlands; ^9^Emergis, Goes, Netherlands; ^10^GGz Delfland, Delft, Netherlands; ^11^Stichting Pameijer, Rotterdam, Netherlands; ^12^Gemeente Rotterdam, Rotterdam, Netherlands; ^13^Department of Clinical Psychology, Vrije Universtiteit, Amsterdam, Netherlands; ^14^Fivoor, Den Haag, Netherlands; ^15^Department of Health Sciences, Hull York Medical School, York Biomedical Research Institute, University of York, York, United Kingdom; ^16^Bavo-Europoort Mental Health Care, Rotterdam, Netherlands

**Keywords:** recovery, personal recovery, psychosis, psychotic disorders, cohort study

## Abstract

Recovery is a multidimensional concept, including symptomatic, functional, social, as well as personal recovery. The present study aims at exploring psychosocial and biological determinants of personal recovery, and disentangling time-dependent relationships between personal recovery and the other domains of recovery in a sample of people with a psychotic disorder. A cohort study is conducted with a 10-year follow-up. Personal recovery is assessed using the Recovering Quality of Life Questionnaire (ReQoL) and the Individual Recovery Outcomes Counter (I.ROC). Other domains of recovery are assessed by the Positive and Negative Symptom Scale Remission (PANSS-R), the BRIEF-A and the Social Role Participation Questionnaire—Short version (SRPQ) to assess symptomatic, functional and societal recovery, respectively. In addition, multiple biological, psychological, and social determinants are assessed. This study aims to assess the course of personal recovery, and to find determinants and time-dependent relationships with symptomatic, functional and societal recovery in people with a psychotic disorder. Strengths of the study are the large number of participants, long duration of follow-up, multiple assessments over time, extending beyond the treatment trajectory, and the use of a broad range of biological, psychological, and social determinants.

## Introduction

**Figure 1:**
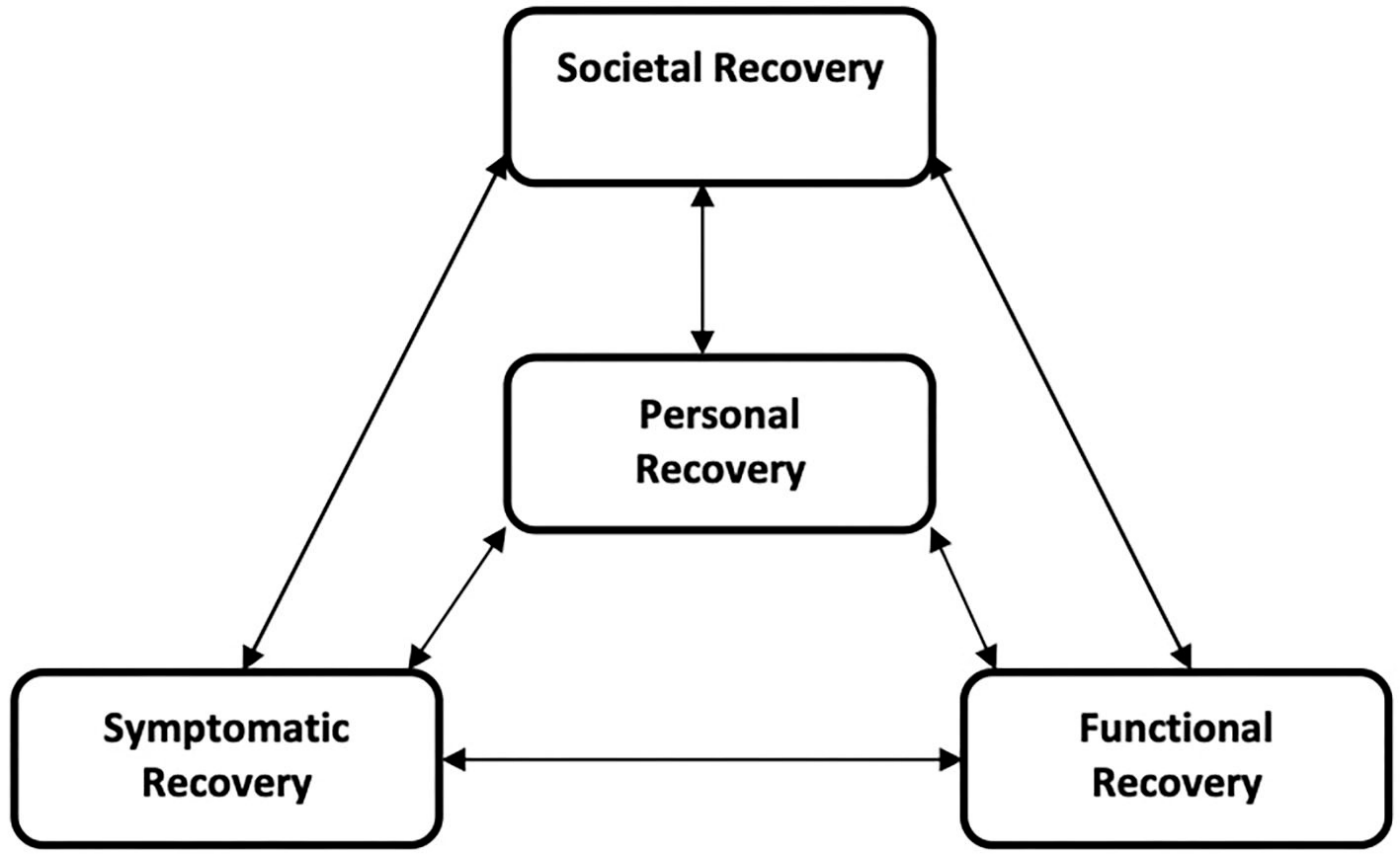
Four dimensional framework of Recovery (2).

The course of personal recovery among people with a psychotic disorder is largely unknown since this a relatively new concept not included in long-term cohort studies. The present study aims to find determinants of personal recovery, and disentangle time-dependent relationships among personal and other dimensions of recovery and its determinants over a 10-year period. Based on Dutch Mental Health Care reports (1–4), recovery can be defined as a four-dimensional framework. This framework consists of personal, symptomatic, societal, and functional recovery, with personal recovery at the center (Figure 1). The dimensions of recovery are thought to be related to one another, but could also be independently achieved (2).

### Personal Recovery

Personal recovery from mental illness is a construct that has gained increased attention over the past thirty years (5). Since the early nineties, participants in the debate on recovery from serious mental illnesses have underlined the notion that personal recovery from a psychotic disorder can occur, either with (6–8) or without psychiatric symptoms being present (9). This is in line with the narratives of people who have experienced mental health issues and used mental health care services (8). It is now acknowledged in mainstream parts of mental health care that mental illnesses should be seen as vulnerabilities, not as a disease that can be cured (10). However, in many studies, those who have remaining symptoms are still considered to be non-recovered (11). Due to the different definitions and ideas used in research and the new line of thought in mental health care, there remains confusion about the concept of personal recovery among clients, their families, clinicians, policy makers and researchers (12). Furthermore, it remains unclear how different factors influence both short- and long-term personal recovery in schizophrenia and other psychotic disorders after treatment (13). There is, however, some consensus on what elements the concept of personal recovery consists of. Publications show that personal recovery should be person-centered, re-authoring, based on exchange, and within the community (14). In line with this analysis, the Slade group stated that (15) personal recovery refers to an individual process of adaptation and development where one does not simply return to, but rather grows beyond the premorbid self.

Slade et al. developed the CHIME conceptual framework for recovery, which identifies five processes that constitute personal recovery: Connectedness, Hope and Optimism, Identity, Meaning in life, and Empowerment (16). However, the CHIME framework has been criticized for being overly optimistic. The difficulties that arise during personal recovery are neglected, as is the influence of therapeutic input. Therefore, the more extensive framework CHIME-D, with the D for “difficulties” (17), has been proposed. However, the specific content of personal recovery, the process is dynamic and non-linear with both gains and relapses (6). A dimension of recovery which should therefore be exclusively client-rated (18).

### Symptomatic Recovery

Symptomatic recovery from psychosis is one of the secondary outcomes in this study, alongside functional and societal recovery. Symptomatic recovery in psychotic illnesses is about reducing positive and negative symptoms (19) using objective, reliable measures (20). A recent analysis showed that symptomatic recovery is constructed from both client-rated and staff-rated factors (18, 21, 22), with health care professionals being leading when deciding which path to take.

### Functional Recovery

Functional recovery focuses on whether someone is capable to recover or compensate for the loss of skills (23). People with schizophrenia may score lower on several dimensions of cognition, like memory, concentration and attention, compared to healthy controls (24, 25), either due to deterioration or impaired development. Especially impairment in executive functions like planning, self-control, and other self-regulating functions (26) can have a major negative impact on one's daily functioning, career, education or social life.

### Societal Recovery

Lastly, societal recovery focuses on counteracting the public stigma on mental illness and improve the position and rights of (ex-)clients within society (20). In this study, we focus on the position of clients in their own social environment by studying how important they find the different social roles and the difficulties they encounter in performing these roles. Furthermore, societal recovery also includes measures on quality of housing, work, education, and social relationships (2). People with SMI often experience difficulties in finding employment and report some form of social exclusion and broken relations with family and friends (27) often due to the stigma of being perceived as dangerous (28–30).

In this four-dimensional framework of recovery, every domain has its own course and characteristics as well as interactions with other domains. Personal recovery is positioned at the center, given it's close connection to the client's own narrative of their own life-course (2). In the present study this multidimensional concept of recovery will be used to try to disentangle the determinants of (the course of) personal recovery.

### Determinants of (Personal) Recovery

Although personal recovery is thought to be the most central and important, the four dimensions may be determined by similar factors. Therefore, we will discuss groups of determinants (i.e., biological, psychological, and social) that may be related to all four dimensions of recovery.

#### Biological Determinants

Age, genetic factors (12, 31), poor physical health, sleep dysfunction, (32) and drug and alcohol use during illness (33) may have an effect on all dimensions of recovery (34–36). These dysfunctions have been linked to poor quality of life (31) and to severity of symptoms (37). The hypothesis is that poor physical health and sleep dysfunction or delay negatively affect the dimensions of recovery. How, and to what extent, these biological factors influence recovery, is dependent on the specific determinant and dimension of recovery.

#### Psychological Determinants

##### Trauma

People with a severe mental illness (SMI) have a higher risk of being traumatized, or become a crime victim (38, 39). For example, both physical and emotional neglect and complex PTSD are highly prevalent in this group (40, 41), especially if a person is also intellectually disabled (42). Furthermore, trauma is thought to have both a direct and indirect negative effect on the onset and symptomatic course of psychotic disorders (43). Therefore, early childhood trauma, recent victimization, and traumatic experiences of the illness itself and during the course of the illness are expected to have a negative impact on all dimensions of recovery.

##### Cognitive Function

Cognition is an umbrella term used to describe multiple mental skills, including learning, memory, problem solving, reasoning, attention, and decision making (44). It is known that impaired cognition is both a predictor for poor outcome in schizophrenia as well as a consequence (45). More specifically, having poor memory is a risk factor for the development of schizophrenia, whereas having a higher verbal IQ is a protective factor (46). During the illness, general cognitive functioning is lower for people with schizophrenia compared to healthy controls (25). Approximately 40% of People with a SMI are suspected to have an intellectual disability (42, 47), with a decreased cognitive flexibility (i.e., mentally switching between, or adapting to different tasks or stimuli) as a consequence. This negatively influences the course of recovery (48). Likewise, illness-insight–partly predicted by cognitive abilities (49)–and treatment compliance (50) are predictors of the course of the illness (50). It is therefore expected that overall cognitive functioning not only predicts better functional recovery, but also better symptomatic and societal recovery.

##### Personality Traits

Psychopathological personality traits can be identified in people with proneness to psychosis (51). These traits are related to the five major categories of personality (i.e., openness, extraversion, conscientiousness, agreeableness, and neuroticism) (52), which are found to influence well-being (53). This goes especially for neuroticism, since adolescents with higher neuroticism scores are at higher risk for developing psychotic symptoms (54, 55). Therefore, it is hypothesized that traits of neuroticism will also negatively affect the process of personal recovery.

##### Empowerment and attachment

Self-esteem, resilience, hopelessness and empowerment (16) are all shown to correlate with Quality of Life measures (56). Furthermore, spirituality, self-esteem (57), attachment (58, 59) and positive social support are thought to contribute to all dimensions of recovery (60). Due to these findings, along with the prominent place empowerment has in the CHIME framework (16), it is believed that the more empowered participants are, the more likely they are to further their personal recovery. Furthermore, the ability to securely attach and thus build personal bonds with those around, is hypothesized to influence personal, functional, symptomatic and societal recovery in a positive manner.

#### Social Determinants

Social capital is thought to be a (health) resource (61), which has cognitive and structural components (62). Cognitive components of social capital are associated with mental health (63) and levels of experienced discrimination (64). The structural part of social capital is derived from social contacts and social participation (65). Social participation thus has an influence on individual health (61), self-reported health (66), and social functioning (67), and is likewise expected to contribute positively to (societal) recovery.

##### Social Factors

Many people with a psychotic disorder suffer from stigmatization and social exclusion (68). For example, public and internalized stigma have been associated with lower levels of perceived social support, recovery, and quality of life (57, 69) and stigma in general is associated with harmed self-esteem (57). Other social factors, including employment and income, contribute to the size of social networks, and the number of social relationships (70), as does living with a partner or as part of a family (71). Clients often define their problems not only in terms of pathology, but also in social terms such as failed friendships, careers, or loneliness (72). Furthermore, employment is positively linked to a health-related quality of life (73). Mental health difficulties like low income, unemployment (74) or poor housing (75) may on the other end act as barriers to social inclusion (76, 77). There is also evidence that suggests migration or ethnic minority status is associated with an inferior social position, which may add to the risk of developing a psychotic disorder (78). Participation and social functioning can thus all influence dimensions of recovery in a positive manner (26), since having one or more social relationships and a wider social network is thought to be critical for achieving recovery (79).

Given all these findings we expect that when high internalized stigma is present, empowerment will be low. Likewise, when there is high internal stigma, recovery—especially personal and societal recovery—will be slower and with more downs. Furthermore, it is hypothesized that when someone is employed, this will positively contribute to societal and personal recovery, as do high(er) income and no debts.

##### Treatment Related Determinants

Evidence-based mental health care with treatment related factors including pharmacological and psychosocial treatments, aim at improving outcome. Therefore, (adherence to) treatment may be an important determinant of outcome. In addition, the number of psychotic relapses and hospitalisations have been associated with outcome.

### Study Objectives

The primary aim of this cohort study is to investigate the proportion of clients who increase on the primary outcome measure of personal recovery over time. Secondary objectives include investigating the proportion of clients who increase on measures of symptomatic, functional and societal recovery over time. Other aims include exploring interactions and time-dependent relationships between the four dimensions of recovery. Furthermore, to explore different effects of determinants, we measure biological (somatic functioning, sleep, drug abuse), psychological (diagnosis, personality traits, anxiety, depression, adverse childhood experiences, trauma, attachment, cognition, social cognition, illness insight, empowerment, resilience), social parameters (internalized stigma and disability in functioning), and treatment related factors (psychosocial treatments and medication). Another secondary objective is identifying how these determinants and their interactions may influence the four dimensions of recovery over time.

Whereas, most studies have focused on only a few episodes of recovery (80), the relatively extensive follow-up period of the current study will make it possible to observe more episodes of recovery over time. Furthermore, most previous research only focused on one aspect or dimension of recovery (81), undermining the complex and multidimensional nature of recovery.

By including all the above-mentioned aspects of a participant's life, this study eventually aims to identify mechanisms which in the end can be translated into (the adaptation of existing) interventions which support clients in their recovery processes.

## Methods/Design

### Study Design

The current study is a multicentre, longitudinal cohort study on recovery from psychotic disorders. The study has a 10-year follow-up period for participants diagnosed with a psychotic disorder.

### Setting

In order to test 600 participants every year, multiple centers in the Netherlands are participating in this study. Every center has multiple facilities, which give care to people with early psychosis and/or reoccurring psychosis. These facilities can either be outreaching or a clinical setting.

### Data Collection

To test all participants, student-researchers will participate in each team and/or facility involved in this study. These student-researchers are MSc student in psychology, medicine or health sciences for whom this study is part of their research internship. They will be trained beforehand and are tasked with informing clients about the study, asking them to participate, including them in the study, interviewing them, processing the data and writing a report for the practitioners. As part of this team, they will sign a confidentially agreement, as is standard in Dutch mental health care. Furthermore, they receive access to the Electronic Patient Files (EPF).

### Recruitment and Consent

Clients will be selected through a search in the EPF of the participating Mental Health Care Institutions. An anonymised list of all clients within a team will be made by the institution. All those not eligible to participate in this study based on the inclusion criteria, will be filtered out. Of the remaining list, 30 clients will be randomly selected. The student-researcher and the team will then receive this list of clients to ask them to participate. Their primary practitioner within the mental health care team is thus aware which clients will be asked to participate and is able to identify those still in active psychosis who are thus unable to participate at that moment. Furthermore, the practitioner can already prepare the client on the visit of the student-researcher, who will be responsible for data collection.

After student-researchers and participants are introduced to one another by the primary practitioner, the first appointment will be made in which the client will be fully informed on the study by the student, and the client will receive the subject information sheet. When all questions are answered, participants are given 2 weeks to consider their participation. If they like to participate, they will be asked to sign an informed consent (IC), of which they will receive a copy. The IC involves consent for feedback to the practitioner, access to the EFP files and using the collected data for research. These are mandatory to participate in the study. Furthermore, the participant is given the option to consent to the study using contacts of choice and using the municipal address database (in Dutch: GBA) when contact through available personal information fails. After the IC is signed, the main health care practitioner will be informed, after which an appointment will be made to include the participant in the cohort. For follow-up assessments, participants are contacted via personal details listed in the EPF. These entail name, surname, maiden name if applicable, date of birth, initials, address, e-mail address and, if consented to, the contact details of two contacts given by the participant. As mentioned earlier, these contact details will only be used if researchers fail to establish contact during follow up, which will most likely be the case when participants are no longer in mental health care or have moved to a different region. Given the aim of the study, those recovering over time are still asked to participate, even though they are referred back to their general practitioner. Likewise, some participants who are worsening over time might lose touch with mental health care. These participants will also be followed-up for the duration of the study. If at any follow-up measurement the participant is unable to participate, e.g., due to active psychosis or incarceration, the measurement will be postponed. However, if a measurement has not been carried out within 9 months, the measurement will fall through and the next measurement will become the objective.

Moreover, measures will be taken to minimize drop-out rates and to increase participant motivation to take part in follow-up interviews. These include sending participants optional birthday cards and regular updates via newsletters and social media channels. Researchers will also regularly visit the cooperating mental health care facilities and participate in any relevant events. Lastly, researchers will regularly evaluate reasons why clients decline participation in order to adjust recruitment strategies accordingly.

### Participants

The research population will consist of clients who receive treatment for a first episode psychosis or have a diagnosis of schizophrenia or another psychotic disorder (schizoaffective disorder, delusional disorder, psychosis not otherwise specified) and who are between 18 and 65 years old. They will be recruited at different Mental Health Care centers in various teams specialized in in- and outpatient care for severe mental illnesses. Furthermore, they all should be able to give informed consent (IC). The aim is to include both clients diagnosed with early psychosis and chronic psychosis. Early psychosis patients are those who have had psychotic symptoms no longer than 5 years. Psychoses are considered chronic, when the psychotic symptoms are present for 5 or more years. Clients who have insufficient proficiency of the Dutch language, will be excluded from participation.

### Measures

#### Primary Outcome Measures

Main study parameter will be scores on two scales assessing personal recovery, measured by the 10-item ReQOL (82) and the I.ROC (83). An overall index score for the ReQOL can be calculated by summing the numbers for the 10 questions where 0 indicates poorest and 40 indicates the highest quality of life. The reliability (internal consistency) in patient samples for the ReQoL-10 is shown to be high (α = 0.85) (82). The I.ROC consists of four dimensions. Three questions are asked on the dimensions of empowerment, home, people and opportunity. The scores range from 0 to 6 per question. The internal consistency is shown to be high (α = 0.86).

Additionally, the Dutch ReQoL-10 will be validated during the course of the study, allowing the construction of normal distribution tables on which cut-off scores for personal recovery will be based.

#### Secondary Outcome Measures

Secondary outcome measures include symptomatic recovery as assessed by the Positive and Negative Symptom Scale–Remission (PANSS-R; α = 0.80) (84). Level of functional recovery will be assessed using the BRIEF-A (self-rated; functional recovery; α = 0.96) (85), and level of social recovery will be assessed using the Social Role Participation Questionnaire [SRPQ; self-rated; social recovery; (86)] over time. The SRPQ has not yet been tested in a psychiatric population, but has been shown to be valid and reliable among patients with Ankylosing Spondylitis (AS) (87). To distinguish levels of symptomatic recovery, the Andreasen remission criterium (88) will be used for the PANSS-R, which states that symptoms are in remission when a score of “mild”/“three” is observed for all eight items, for 6 months. The PANSS-R will only be administered every year, so it will be necessary to deviate from the time threshold of 6 months. Cut-off scores for functional and social recovery will be based on normal distribution tables from the BRIEF-A (89) and the SRPQ, respectively, in which (sub)clinical levels of functional and social recovery can be identified. Psychiatric history and psychiatric diagnosis will be assessed using the OPCRIT+ electronic tool (90).

#### Determinants

##### Biological Determinants

Somatic health will be assessed using the Physical Complaints Questionnaire (in Dutch: *Lichamelijke Klachten Vragenlijst*-51) (91) and the Treatment Inventory of Costs in Patients with psychiatric disorders (TIC-P; α= 0.83) (92). Subsequently, sleep (patterns) will be measured through the Pittsburgh Sleep Quality Index (PSQI; α= 0.83) (93). Furthermore, substance abuse will be assessed using the Addictions for Triage and Evaluations questionnaire (MATE; α= 0.75–0.92) (94). A standard physical examination (PE) is done every year in people with both early and chronic psychosis. During these physical checks, length, weight, abdominal girth and blood pressure are measured. Furthermore, a case report form containing questions about age, gender, medication, lifetime psychiatric illness diagnosis and psychiatric history (including number of compulsory admissions) will be filled in as well.

##### Psychological Determinants

Intellectual disability will be screened using the *Screener for Intelligence and Learning Disabilities (SCIL)*, which has been shown to have good internal consistency (Cronbach's α= 0.83) (95). Depression and anxiety will be screened using the Patient Health Questionnaire-9 (PHQ-9; α = 0.89) (96) and Generalized Anxiety Disorder-7 (GAD-7; α = 0.92) (97) screening scales. Impaired cognition will be assessed using the Brief Assessments Cognition in Schizophrenia (BACS; α > 0.79 for all subtests) (24) and the social cognitive tasks will be the Picture Arrangements (98, 99) and the Hinting Task (100). Furthermore, personality traits, trauma, early childhood trauma and attachment will be assessed using the NEO-Five Factor Inventory (NEO-FFI; α = .88) (101), Trauma Screening Questionnaire (TSQ; AUC = 0.85) (41), Adverse Childhood Experience questionnaire (ACE; α = 0.76) (102, 103)and the Psychosis Attachment Measure (PAM; attachment anxiety α = 0.82 and attachment avoidance α = 0.76) (104), respectively.

Psychological processes like coping, resilience, empowerment, insight and therapy compliance will be assessed over time with the Coping Inventory in Stressful Situations (CISS; α= 0.86) (105, 106), Brief Resilience Scale (BRS; α = 0.83) (107, 108), Dutch Empowerment List (NEL; α = 0.94) (89), Brief Cognitive Insight Scale (BCIS; α = 0.60–0.68) (109) and Therapy adherence Scale (SES; α = 0.91) (110). Internalized stigma will be assessed using the ISMI-10 (α = 0.94) (111, 112).

##### Social Determinants

Social factors like education, work and work-history will be registered, along with administering the World Health Organization—Disability Assessment Schedule 2.0 (WHO-DAS 2.0; α = 0.95) over time (113). Basic social capital demographics, family history, socio-economic status, living situation, family composition, working situation, and income will also be enquired.

#### Assessment of Treatment Related Factors

Prescribed medication and psychosocial treatments will be assessed form the electronic patient files.

### Questionnaires and Instruments

An overview of all the measurements is given in Table 1. The time for each questionnaire is also listed. When questionnaires are filled in afterwards by the researcher, and thus does not involve the participants involvement, the time is listed between brackets. All primary and secondary outcome measures will be assessed every year. All other questionnaires and procedures will be repeated every 2 years, with the last measurement 10 years after baseline. Table 2 shows which questionnaires will be assessed over time. All questionnaires and procedures baseline will be assessed through a face-to-face interview at the participants home or at the health-care institution. Follow-up measurements can be done either face-to-face, over the phone, or via a combination of both. In both instances, the questionnaires can be spread out over multiple appointments in order to make sure the participant is able to answer all questions as is, without weariness or diminished attention getting in the way. Furthermore, this set-up also decreases the burden of the long and vast interview. Both the number of appointments and the location of the appointments are dependent on the preference of the participant. In some cases, face-to-face interviews in which the questionnaires will be read out loud and explained will be necessary and mandatory. This can be the case for e.g., participants with a possible learning disability. Student-researchers are trained to do so without changing the content of the questionnaires and without nudging participants to a certain answer.

**Table 1:** Questionnaires and instruments.

**Parameters**		**Test**	**Time (min)**
Primary	Personal	Recovering quality of life, short version 10 items (self-rated).	5
outcome	recovery	Individual recovery outcome counter (self-rated)	10
Secondary	Symptomatic recovery	Positive and negative symptoms scale—remission (observer-rated)	10
Outcomes	Functional recovery	Behavior rating inventory of executive function—adult version (self-rated)	15
	Societal recovery	Social role participation questionnaire—short form (self-rated)	10
Determinants	Biological	Physical complaints questionnaire 51 (in Dutch: Lichamelijke klachtenvragenlijst 51; self-rated)	[30] 15
		Questionnaire on healthcare utilization and productivity losses in patients with a psychiatric disorder (self-rated)	10
		Pittsburgh sleep quality index (self-rated)	5
	Psychological	Opcrit+	[45]
		Screener for Intelligence and mental challenge (in Dutch: screener voor intelligentie en licht verstandelijke beperking, SCIL; observer-rated)	10
		NEO—five factor inventory (self-rated) Patients Health Questionnaire—Depression Scale (self-rated) Generalized anxiety disorder—anxiety scale (self-rated)	10 5 3
		Adverse childhood experience (observer rated)	3
		Trauma screening questionnaire (self-rated)	5
		Measurements in the addictions for triage and evaluations part 1 & 4 (observer rated)	10
		Psychosis attachment measure (self-rated)	10
		Brief assessment of cognition in schizophrenia (observer-rated)	35
		Social cognitive tests (observer-rated) - Picture arrangements - Hinting task	21 10 11
		Coping inventory for stressful situations (self-rated)	10
		Beck cognitive insight scale (self-rated)	10
		Netherlands empowerment list—short version (in Dutch: Nederlandse empowerment lijst; self-rated)	10
		Brief resilience scale (self-rated)	3
		Internalized stigma of mental illness, short version (self-rated)	10
	Social	WHO-DAS 2.0 (observer-rated)	20
Other		Routine monitoring outcome (self-rated)	[15]
		Mental health—cluster index test (In Dutch: GGZ kluster indicatoren toets; observer rated)	[15]
		Integrated recovery list (in Dutch: Integrale herstel lijst; observer/self- rated)	[15]
		Service engagement scale	[15]
		Treatment overview (including medication)	[30]

**Table 2:** Overview of the procedures per time point.

**Year**	**0**	**1**	**2**	**3**	**4**	**5**	**6**	**7**	**8**	**9**	**10**
ReQOL	X	X	X	X	X	X	X	X	X	X	X
I.ROC	X	X	X	X	X	X	X	X	X	X	X
PANSS	X	X	X	X	X	X	X	X	X	X	X
BRIEF-A	X	X	X	X	X	X	X	X	X	X	X
SRPQ - SF	X	X	X	X	X	X	X	X	X	X	X
LKV-51	X		X		X		X		X		X
TiC-P	X		X		X		X		X		X
PSQI	X		X		X		X		X		X
OPCRIT+	X										
SCIL	X										
NEO-FFI	X										
PHQ-9	X		X		X		X		X		X
GAD-7	X		X		X		X		X		X
ACE	X										
TSQ	X		X		X		X		X		X
MATE	X		X		X		X		X		X
PAM	X		X		X		X		X		X
BACS	X		X		X		X		X		X
SCT	X		X		X		X		X		X
CISS	X		X		X		X		X		X
BCIS	X		X		X		X		X		X
NEL	X		X		X		X		X		X
BRS	X		X		X		X		X		X
ISMI-10	X		X		X		X		X		X
WHO-DAS	X		X		X		X		X		X
ROM	X	X	X	X	X	X	X	X	X	X	X
GGZ KIT	X	X	X	X	X	X	X	X	X	X	X
IHL	X	X	X	X	X	X	X	X	X	X	X
SES	X		X		X		X		X		X
PE	X	X	X	X	X	X	X	X	X	X	X
BT	X	X	X	X	X	X	X	X	X	X	X

*ReQOL, Recovering Quality of Life; I.ROC, Individual Recovery Outcome Counter; PANSS-R, Positive and Negative Symptom Scale—Remission; BRIEF-A, Behavior Rating Inventory of Executive Function—Adult Version; SRPQ—SF, Social Role Participation Questionnaire—Short Form; LKV-51—Physical Complaints Questionnaire; TiC-P, Questionnaire on healthcare utilization and productivity losses in patients with a psychiatric disorder; SCIL, Screener for Intelligence and Mentally Challenged; PHQ-9, Patients Health Questionnaire—Depression Scale; GAD-7, Generalized Anxiety Disorder—Anxiety Scale; NEO-FFI, NEO Five Factor Inventory; ACE, Adverse Childhood Experiences; TSQ, Trauma Screening Questionnaire; MATE, Measurement in the Addictions for Triage and Evaluations; PAM, Psychosis Attachment Measure; BACS, Brief Assessment of Cognition in Schizophrenia; SCT, Social Cognitive Tasks; CISS, Coping Inventory for Stressful Situations; BCIS, Beck Cognitive Insight Scale; NEL, Netherlands Empowerment List; BRS, Brief Resilience Scale; ISMI-10, Internalized Stigma of Mental Illness, short version; WHO-DAS 2, World Health Organization—Disability Assessment Schedule 2.0; SES, Service Engagement Scale; ROM, Routine Outcome Monitoring; GGZ KIT, Mental Health—Cluster Index Test; IHL, Integrated Recovery List; PE, Physical Examination; BT, Blood Test; Medication, overview of taken medication in the last year; Treatment, overview of received treatment in the last year*.

Furthermore, a yearly physical examination (length, weight, abdominal circumference and blood pressure) and blood test (Hemoglobin, leukocytes and differentiation, renal function, liver function, triglycerides, HDL-cholesterol, prolactin, glucose) are gathered from the EPF. Since these are standardized care in this patient population in the Netherlands, and participants consent to gathering this information via their general practitioner while they are still receiving care. Participants receive a compensation of 25 euros for each completed measurement.

#### Observations and Assessment Results

Observations and/or assessment results might be relevant for current care. Furthermore, some topics might reveal thoughts or actions which can be harmful to the participant or their environment, but which are not known by the practitioner. Therefore, all results are reported back to the mental health care team which gives care to the participant.

### Patient Involvement

Service users/experience experts are involved in the design of the study, development and implementation of study protocols, and monitoring the study process. A group of peer-experts has been consulted during the process of creating this cohort. They made sure the interest and the vision of the process of recovery from clients would be valued and integrated in this cohort. Additionally, a small pilot study has been done with five peer-expert students to test the length and order of the interview and get their feedback on the questionnaires themselves. The results have been discussed with the original group of peer-experts, and changes to the organization of the interview were made in cooperation with these peer-experts. Furthermore, experience experts are present during the training of the student-researchers, guiding them through the meaning of recovery and letting them experience the concept, but also helping them streamline the interview in a way that is least demanding for the participants but without changing the validity of any of the questionnaires.

Furthermore, service users/experience experts attached to participating teams will support the student-researchers in developing a good rapport and giving the feedback to the participants.

### Sample Size Calculation

Personal recovery rates in schizophrenia and other psychotic disorders are estimated to be about 14% (80). We expect to observe recovery over time in a minimum of 10% of all subjects over a period of 10 years and opt for a desired precision of 5%. Precision-based sample size calculation using the formula: *n* = (Z^2^ × P(1–P))/e^2^ (where *Z* = 1.96 for 95% CI, P is expected true proportion, and e is half the desired precision), results in 553 participants. Expected loss to follow-up is estimated at about 15% which gives a total of 651 participants to include.

### Statistical Analysis

Primary analysis is to estimate the proportion of patients achieving personal recovery over a period of 10 years. The main focus will be on the interrelation of domains of recovery and impacts of different factors on the course of recovery. Using generalized linear mixed models, repeated measures of ReQOL and I-ROC will be the dependent variables, time will be the within-subjects factor and the biological, psychological and social determinants will be independent variables. All parameters will be checked for outliers; data will be transformed when necessary. Missing values will be dealt with accordingly. All (114), detailed descriptions will be given of participants included in each analysis.

## Results

### Primary Outcome

For our primary analysis, we will calculate the proportion of clients who reach personal recovery as measured by the ReQOL and I-ROC questionnaires. Initial analysis will be based on dichotomized scores without covariates. In additional analyses, we model personal recovery over time. Using generalized linear mixed models, repeated measures of ReQOL and I-ROC will be the dependent variables, time will be the within-subjects factor and the biological, psychological, and social determinants will be independent variables.

### Secondary Outcome

To investigate the associations between personal, clinical, societal and functional recovery, analyses will focus on changes in PANSS, BRIEF-A and SRPQ scores over time, respectively. Generalized linear mixed models will be fit to explore the course of recovery in relation to other recovery dimensions as time-dependent co-variates.

## Discussion

This study aims to assess the course of personal recovery, and to find determinants and time-dependent relationships with symptomatic, functional, and social recovery in persons with a psychotic disorder.

Participants with a psychotic disorder, from multiple healthcare centers in The Netherlands, will be interviewed every year for 10 years. Furthermore, biological, psychological and social determinants that in previous research have been associated with the four dimensions of recovery will be measured over time. Ultimately, this study hopes to contribute to the understanding of the complexity of recovery. In this study we hope to unfold time-dependent relationships between different forms of recovery and its determinants. Thus, (new) interventions can be tailored to better fit the needs of people who suffer from a psychotic disorder, so that recovery will be possible for many more of these people.

### Strengths and Limitations

Strengths of the study are the large number of participants, long duration of follow-up, multiple assessments over time (also when participants are not in mental health care anymore), and the use of a broad range of biological, psychological, and social determinants. The entire study has been excogitated with the help of both a scientific board, consisting of established researchers from the different participating mental health care institutions, and a group of service users/experience experts.

Limitations are that, since this is an observational study, it is not intended to study intervention effects. However, the relatively long follow-up period is expected to provide information on time-dependent relationships. Secondly, two-yearly assessments of secondary measures will not capture all variation details over time. In add-on studies we hope to zoom in on the course of these outcomes in selected sub-samples. Thirdly, despite random selection of clients meeting the criteria, care avoiding or less motivated clients may not be willing to participate, possibly skewing our study population toward a more positive picture of the process of recovery. Fourth, the inability to include people not fluent in Dutch will limit the generalisability of this study. As mentioned, those with a migration status might have major social disadvantage, possibly causing the process of recovery to be very different and more difficult. By excluding those not able to answer the questionnaires due to language, we are unable to get the full picture of the process for that group. Fifth, this cohort does not take the recovery orientation per institution or team into account. We are therefore unable to control for the degree of recovery orientation. However, participants will be clustered within teams. Team-level might thus reflect whether team differences—like recovery orientation or other team-related or organizational differences—are of influence on the process of recovery for participants.

Lastly, all assessments mentioned above are assessments on topics which are known to be important in the course of the illness. Therefore, it is believed that a lot of these topics have been discussed to some extent with the practitioner, but have not (always) been objectified in a formal, numerical manner. Thus, although these interviews are observational, practitioners may influence some clinical decisions on the information gathered from the study.

## Ethics Statement

The studies involving human participants were reviewed and approved by Medisch Ethische Toetsingscommissie Erasmus MC Rotterdam. The patients/participants provided their written informed consent to participate in this study.

## Author Contributions

BA and ABa have written this protocol, under the direct guidance of AW and CM. All authors have commented on multiple versions of this manuscript. All authors have read and approved the final manuscript.

## Conflict of Interest

The authors declare that the research was conducted in the absence of any commercial or financial relationships that could be construed as a potential conflict of interest.

## Abbreviations

ACE: Adverse Childhood Experience
BACS: Brief Assessment of Cognition in Schizophrenia
BCIS: Birchwood Cognitive Insight Scale
BRIEF-A: Behavior Rating Inventory of Executive Function—Adult version
BRS: Brief Resilience Scale
BT: Blood Test
CISS: Coping in Stressful Situations
EPD: Electronic Patient Dossier
GAD-7: Generalized Anxiety Disorder−7-item Anxiety Scale
GGZ KIT: Mental Health—Cluster Index Test (In Dutch: GGZ Kluster Indicatoren Toets)
IC: Informed Consent
I.ROC: Individual Recovery Outcome Counter
IHL: Integrated Recovery List
ISMI: Internalized Stigma of Mental Illness
LKV: Bodily Complaints Questionnaire
MATE: Measurements in the Addiction Triage and Evaluation
NEL: Netherlands Empowerment List
NEO—FFI: NEO Five Factor Inventory
PAM: Psychosis Attachment Measure
PANSS—R: Positive and Negative Symptoms Scale—Remission
PHQ-9: Patients Health Questionnaire—Depression Scale
PIF: Patient Information Form
PSQI: Pittburgh Sleep Quality Index
ReQOL: Recovering Quality of Life
ROM: Routine Outcome Monitoring
SCIL: Screener for Intelligence and Mental Challenge (in Dutch: Screener voor Intelligentie en Licht verstandelijke beperking)
SCT: Social Cognitive Tasks
SEL: Service Engagement Scale
SRPQ—SF: Social Role Participation Questionnaire—Short Form
TiC-P: Questionnaire on healthcare utilization and productivity losses in patients with a psychiatric disorder
TSQ: Trauma Screening Questionnaire
WHO-DAS: World Health Organization—Disability Assessment Schedule
ACE: Adverse Childhood Experience
BACS: Brief Assessment of Cognition in Schizophrenia
BCIS: Birchwood Cognitive Insight Scale
BRIEF-A: Behavior Rating Inventory of Executive Function—Adult version
BRS: Brief Resilience Scale
BT: Blood Test
CISS: Coping in Stressful Situations
EPD: Electronic Patient Dossier
GAD-7: Generalized Anxiety Disorder−7-item Anxiety Scale
GGZ KIT: Mental Health—Cluster Index Test (In Dutch: GGZ Kluster Indicatoren Toets)
IC: Informed Consent
I.ROC: Individual Recovery Outcome Counter
IHL: Integrated Recovery List
ISMI: Internalized Stigma of Mental Illness
LKV: Bodily Complaints Questionnaire
MATE: Measurements in the Addiction Triage and Evaluation
NEL: Netherlands Empowerment List
NEO—FFI: NEO Five Factor Inventory
PAM: Psychosis Attachment Measure
PANSS—R: Positive and Negative Symptoms Scale—Remission
PHQ-9: Patients Health Questionnaire—Depression Scale
PIF: Patient Information Form
PSQI: Pittburgh Sleep Quality Index
ReQOL: Recovering Quality of Life
ROM: Routine Outcome Monitoring
SCIL: Screener for Intelligence and Mental Challenge (in Dutch: Screener voor Intelligentie en Licht verstandelijke beperking)
SCT: Social Cognitive Tasks
SEL: Service Engagement Scale
SRPQ—SF: Social Role Participation Questionnaire—Short Form
TiC-P: Questionnaire on healthcare utilization and productivity losses in patients with a psychiatric disorder
TSQ: Trauma Screening Questionnaire
WHO-DAS: World Health Organization—Disability Assessment Schedule.
