# Corrigendum: UP'S: A Cohort Study on Recovery in Psychotic Disorder Patients: Design Protocol

**DOI:** 10.3389/fpsyt.2021.771914

**Published:** 2021-10-19

**Authors:** Bernice C. van Aken, Ayuk Bakia, André I. Wierdsma, Yolande Voskes, Jaap Van Weeghel, Evelyn M. M. van Bussel, Carla Hagestein, Andrea M. Ruissen, Pien Leendertse, Wishal V. Sewbalak, Daphne A. van der Draai, Alice Hammink, M. E. Mandos, Mark van der Gaag, Annette E. Bonebakker, Christina M. Van Der Feltz-Cornelis, Cornelis L. Mulder

**Affiliations:** ^1^Department of Psychiatry, Erasmus MC, Epidemiological and Social Psychiatric Research Institute, Rotterdam, Netherlands; ^2^Department of Ethics, Law and Humanities, Amsterdam UMC, Amsterdam, Netherlands; ^3^GGz Breburg, Tilburg, Netherlands; ^4^Phrenos Centre of Expertise, Utrecht, Netherlands; ^5^Tranzo Department, Tilburg School of Behavioural and Social Sciences, Tilburg University, Tilburg, Netherlands; ^6^Parnassia Psychosis Research, Den Haag, Netherlands; ^7^GGz Oost Brabant, Oss, Netherlands; ^8^Antes, Rotterdam, Netherlands; ^9^Emergis, Goes, Netherlands; ^10^GGz Delfland, Delft, Netherlands; ^11^Stichting Pameijer, Rotterdam, Netherlands; ^12^Gemeente Rotterdam, Rotterdam, Netherlands; ^13^Department of Clinical Psychology, Vrije Universtiteit, Amsterdam, Netherlands; ^14^Fivoor, Den Haag, Netherlands; ^15^Department of Health Sciences, Hull York Medical School, York Biomedical Research Institute, University of York, York, United Kingdom; ^16^Bavo-Europoort Mental Health Care, Rotterdam, Netherlands

**Keywords:** recovery, personal recovery, psychosis, psychotic disorders, cohort study

In the original article, reference (100) was incorrectly written as “de Sonneville L. Amsterdamse neuropsychologische taken: wetenschappelijke en klinische toepassingen. *Tijdschr voor Neuropsychol*. (2005) 10:27–41”.

It should be “Corcoran R, Mercer G, Frith CD. Schizophrenia, symptomatology and social inference: investigating “theory of mind” in people with schizophrenia. *Schizophr Res*. (1995) 17:5–13.”

The authors apologize for this error and state that this does not change the scientific conclusions of the article in any way. The original article has been updated.

## Publisher's Note

All claims expressed in this article are solely those of the authors and do not necessarily represent those of their affiliated organizations, or those of the publisher, the editors and the reviewers. Any product that may be evaluated in this article, or claim that may be made by its manufacturer, is not guaranteed or endorsed by the publisher.

## References

[100] Corcoran R Mercer G Frith CD. Schizophrenia, symptomatology and social inference: investigating “theory of mind” in people with schizophrenia. Schizophr Res. (1995) 17:5–13. 8541250 10.1016/0920-9964(95)00024-g

